# The reimbursement decision speed for oncology new drugs in China and its determinant factors

**DOI:** 10.3389/fpubh.2023.1207739

**Published:** 2023-10-30

**Authors:** Xingyue Zhu, Yang Chen

**Affiliations:** ^1^Department of Pharmacy Administration, School of Medicine and Health Management, Guizhou Medical University, Guiyang, Guizhou, China; ^2^The Third People's Hospital of Chengdu, Chengdu, Sichuan, China

**Keywords:** reimbursement, time to list, price negotiation, tradeoff, drug access

## Abstract

**Introduction:**

China has initiated national price negotiations to improve access to innovative drugs. Learning the factors that contributed to the time gap from marketing authorization to reimbursement leads to more clarity to decision-making, which remains under-researched in China.

**Methods:**

We collected new oncology drug approvals that were marketed before 30 Jun 2022, using the Listed Drug Database of the Chinese drug agency. Major information of each approval was obtained from the published review report, including the first approval region (China or the US) and the receipt of expedited review pathways (priority review and conditional approval). The reimbursement lists issued by China National Healthcare Security Administration from 2015 to 2023 were used to determine the reimbursement status of drugs. The duration from marketing authorization to reimbursement was defined as the reimbursement decision speed, and the Cox regression was performed to explore the underlying factors.

**Results:**

A total of 186 oncology approvals were included. More than half of the approvals qualified for reimbursement (110[59.14%]), and the median reimbursement decision speed was accelerated from 540.5 days in the third-round negotiation to 448 days in the seventh-round. Domestic new drugs had a higher probability of being adopted by the Chinese payer than drugs developed by foreign companies (adjusted *HR* = 3.73, 95% CI 2.42 to 5.75; *P* < 0.001). Furthermore, new drug applications receiving the regular review pathway were more likely to be reimbursed (adjusted *HR* = 2.15, 95% CI 1.13 to 4.08; *P* = 0.020) compared to those approved under the conditional approval pathway.

**Discussion:**

These findings indicate that the Chinese government is actively working toward improving access to new oncology drugs. The faster reimbursement decision speed for domestic drugs might be attributed to their pricing advantages and the regulator's efforts to stimulate innovation in the domestic pharmaceutical industry. However, concerns about the uncertainty in drug benefits can affect the reimbursement decision-making, which suggests the delicate tradeoff between drug accessibility and risk involved in the reimbursement process.

## 1. Introduction

China used to suffer from serious drug lag, which precluded patients from using state-of-the-art technologies to improve their health in a timely manner (1–3). This issue was related to the redundant drug review process, the lack of interest from global developers in expanding their business in China, and the weak domestic pharmaceutical industry (3–5). In the meantime, government underfunding, lax drug price regulation, and infrequent updates to the reimbursement list for the public health insurance system, known as the China Basic Medical Insurance (BMI), led to a dearth of coverage for most novel pharmaceuticals in the public plan (6, 7). The limited access to and prohibitive out-of-pocket cost of new drugs were among the key issues contributing to the widespread public dissatisfaction with the health system (8). To promote the accessibility of new drugs, in 2015, China rolled out initiatives in the drug regulatory system toward faster delivery of new drugs with high clinical interests, based on which expedited review pathways were launched (9). These are primarily the priority review pathway and the conditional approval pathway (10). Priority review is established to abbreviate the review length, while conditional approval grants early approval on surrogate endpoints with the mandatory requirement for a post-marketing confirmatory study (Supplementary Table 1). In the same year, national price negotiation was first introduced as a new approach for high-priced branded drugs to be eligible for reimbursement (11). As a consequence, three drugs ended up in the negotiation with price drops of over 50% (12), and the formulary of BMI began to be updated after a 7-year stagnation. The price negotiations are conducted by the government, currently led by the Chinese payer, the National Healthcare Security Administration (NHSA). These negotiations are carried out under the value assessment framework. Cost-effectiveness analysis and budget impact analysis are the primary bases for bargaining (13). From 2015 to January 2023, there have been seven rounds of price negotiations. The first three rounds (respectively, held in 2015, 2017, and 2018) were exploratory, with no fixed implementation frequency, and all negotiation candidates were determined by expert groups (14). Since the fourth round (held in 2019), negotiation has been implemented in the latter half of each year, with the resultant new reimbursement taking effect the following year, and manufacturers are able to apply to participate in the negotiations. The current workflow of price negotiation consists of four consecutive stages: first, NHSA formulates the annual working plan; second, companies submit applications according to the working plan; third, NHSA carries out desk reviews to select applications concordant with the requirement and forms the list of candidates qualifiable for negotiation; and finally, the third-party experts invited by NHSA conduct negotiations with the companies based on economic evaluation results to reach a mutually acceptable price point (15). With the mounting use of expedited review pathways (16), an increase in expedited drugs is expected in price negotiations, thereby facilitating faster and more affordable drug access. Nonetheless, the effects of expedited review pathways on the reimbursement decision have received little research attention.

In addition to refining drug access, another important task of these reform efforts is to encourage innovation, particularly in the domestic pharma industry (17). Relying on the longer profitable window phase via earlier market entry enabled by expedited pathways, as well as the expanded market size after reimbursement, the pharma companies are anticipated to generate more revenue so as to further invest in products with the promises of superiority over available therapies and of fulfilling unmet medical needs. Accordingly, these days NHSA is tasked with providing individual financial protection, optimizing the use of limited funds, and stimulating innovation. Well-tailored strategies are important for the payer to meet its multiple objectives, and the first step is to learn the payer's considerations in its decision making process. Previous research on factors affecting reimbursement decision-making has yielded mixed and inconclusive results due to their varying study designs and contexts (18–21). Moreover, China's oncology pharmaceutical market is experiencing a significant upsurge, and some Chinese pharma companies are now even specializing in oncology therapies (22). In this context, many home-grown anti-cancer drugs have been delivered to patients and have accessed BMI through aggressive pricing strategies in price negotiations (23, 24). Furthermore, cancers are seriously life-threatening conditions with urgent unmet medical needs, and thus novel drugs treating cancers can usually benefit from regulatory incentives aimed at addressing unmet medical needs. To some extent, the oncology pharmaceutical industry is the most responsive to policy evolution, including the refined review system and the innovative drug reimbursement policy. Whether domestic oncology products undergo different reimbursement decision speeds as a response to the regulator's target to encourage domestic innovation, or as a consequence of more favorable cost-effectiveness, also remains unknown. In this study, we aim to delve into the factors influencing drug reimbursement decision speed in China based on the data on new oncology drugs. Our study will help further understand the payer's decision-making and shape the basis for future research and actions to ensure timely, safe, and sustainable access to new drugs.

## 2. Methods

### 2.1. Data

The Listed Drug Database, an open database established by the China National Medical Product Administration (NMPA), provides access to the review report and approved label of drug approvals (25). This database was set up recently, and so far it only includes the drug approvals licensed from 2015 onwards. In addition, due to its ongoing construction, there are some historical approvals pending publication. Based on the Listed Drug Database, we collected all the new oncology approvals (new molecular entities and biologics) that were marketed before 30 June 2022. This timeframe was set to accord with the eligibility criteria of the most recent negotiation (the seventh round).

The factors of interest pertaining to drug characteristics included (1) registration class, which was classified as either new drug application (NDA) for new molecule entities or biologics license application (BLA) for biologics, respectively; (2) approval class, which was categorized as either the marketing approval for the initial license for a drug or the new indication supplement for any supplementary approval for a marketed drug's new approved indication; (3) year of approval; (4) review times, defined as the calendar dates from the submission date to the approval date; (5) priority review designated by NMPA; (6) conditional approval designated by NMPA; (7) cancer site based on the approved indication, which was broken down into 11 sites of lung, hematologic, alimentary system, breast, genitourinary, gynecological, skin, head and neck, mixed solid tumors, thyroid, and others; (8) region of first approval, which was used to identify whether a specific drug's first approval was in China, with the US serving as the reference country for determining the first approval region. The variable, the region of first approval, was actually an alternative for domestic-leading research and development (R&D). Since one major question of our interest was whether domestic drugs experienced different reimbursement decision speeds, it was important to determine the drugs developed by Chinese companies. However, the nationality of the sponsor was not a good identifier because some foreign new drugs were out-licensed to Chinese companies in order to obtain market authorization in China. On this account, we used the region of first approval instead. We assumed that drugs developed by domestic companies would prioritize the Chinese market, while out-licensed drugs were more likely to have already obtained approval outside of China, such as in the home country of the original foreign developers.

Information on all the above variables was assembled from the disclosed review report for each approval. We used the reimbursement lists from the previous seven rounds of price negotiation to ascertain the drugs eligible for BMI coverage, which were available on the government website (www.gov.cn). The seventh negotiation was finished in January 2023, according to which the latest reimbursement list has been in effect since 1 March 2023. Hence, the reimbursement status for the included approvals was set to be followed up to 1 March 2023.

### 2.2. Statistical analysis

We used descriptive statistics to characterize the included approvals. The reimbursement decision speed, defined as the elapsed time from marketing approval to reimbursement, formed the time-to-event outcome. Thus, we utilized the Cox proportional-hazards regression to conduct the multivariate analysis, in which the Wald test was used for statistical inference. The main factors of interest were the first approval region, along with priority review designation and conditional approval designation. Other covariates included in the Cox model were registration class, approval class, review times, and approved year. We did not include cancer sites in the Cox model, since we were concerned that introducing such a categorical variable with many levels in a relatively small sample would lead to overspecification. More importantly, we argued that some drug features had a greater influence on regulatory decision-making than cancer sites, such as disease prevalence and therapeutic value magnitude. The orphan designation was supposed to be a good identifier for these important drug-specific features. Nonetheless, China has not established an orphan designation, which makes it difficult to identify rare diseases and introduces certain limitations to our findings. In the model, approved years were rearranged into two classes of <2020 or ≥2020 to reduce our concern about overspecification as well. Given that our study drugs covered the period from 2015 to 2022, we used 2020 as the cutoff point to divide the study period evenly. To further delve into the effects of one specific variable among drugs with different features of another variable, an interaction analysis between the two variables will be conducted. For example, to ascertain whether the conditional approval pathway would have different effects among NDAs and BLAs, the interaction between conditional approval and registration class would be needed. Moreover, the fourth and subsequent rounds of price negotiations allow unsolicited applications from manufacturers. As such, our sample of all the marketed oncology drugs might introduce confounding due to the fact that some manufacturers had no intention of accessing the national coverage plan during the study timeframe. NHSA released the lists of negotiation candidates that had applied for negotiations and passed the desk review, based on which we conducted the subgroup analysis for all drugs entering into the negotiations. The significance level was set to be 0.05 for two-tailed tests, and robust standard errors were used. Stata version 15 (StataCorp LP) was used to perform the analysis.

## 3. Results

### 3.1. Features of the approvals studied

**Figure 1:**
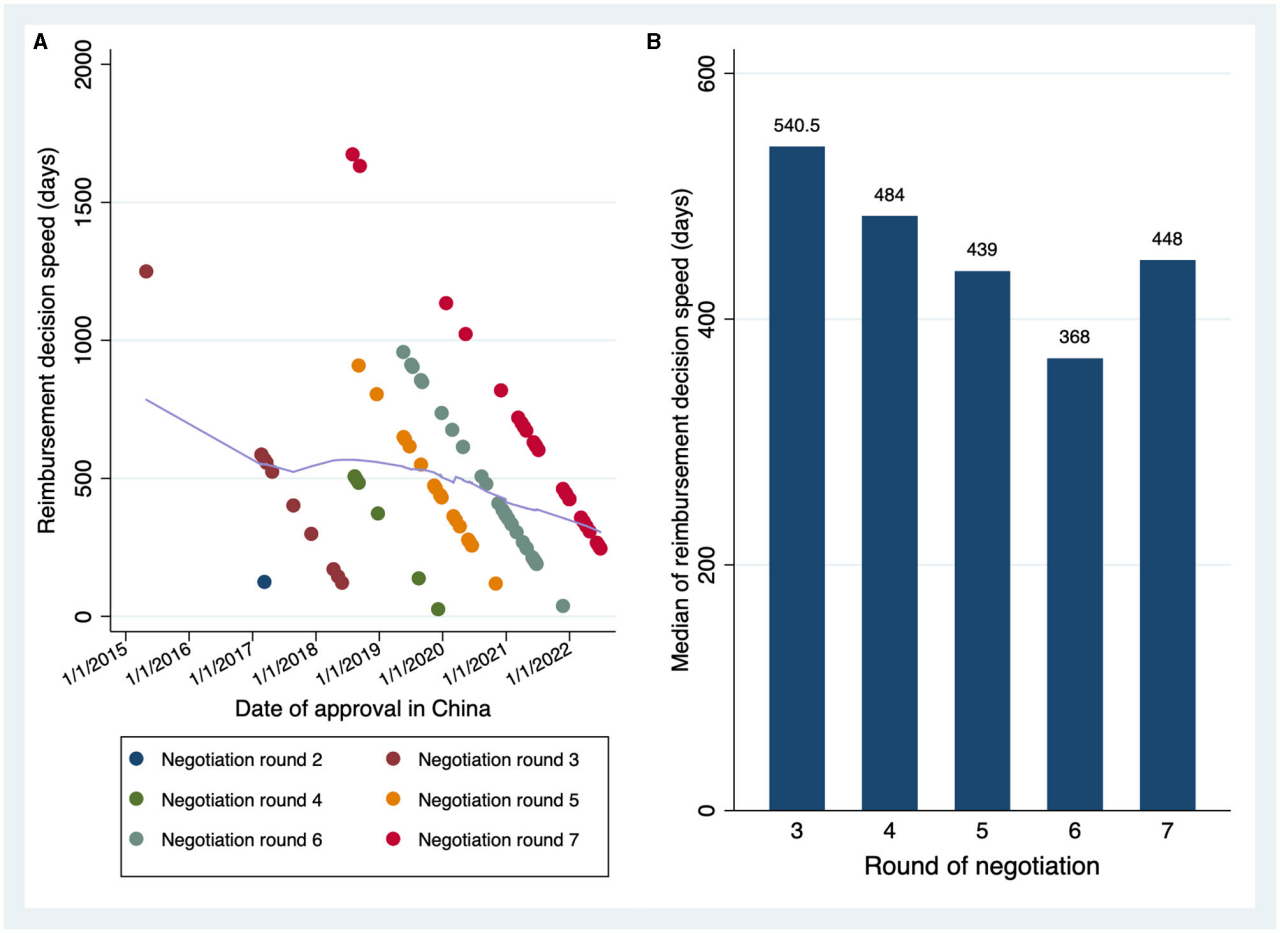
The trend of the reimbursement decision speed. **(A)** Reimbursement decision speed in terms of drug approval timings. Scatters in different colors denoted the drugs winning the different rounds of negotiation. The fitted line in light purple was constructed by the locally weighted scatterplot smoothing (LOWESS) approach, which indicates the tendency of the time to reimbursement over time. **(B)** Reimbursement decision speed in terms of negotiation rounds.

A total of 186 oncology approvals were determined (Table 1). NDAs [102 (54.84%)] accounted for a slightly higher proportion of the total than BLAs [84 (45.16%)]. Half of the approvals were post-marketing new indication supplements [93 (50%)]. Lung cancer was the most common indication [44 (23.66%)], followed by hematologic malignance [39 (20.97%)]. The majority of approvals were licensed in 2020 and beyond [131 (70.43%)]. Additionally, a great number of drugs were developed and first approved outside China [112 (60.22%)]. Less than 40% of the drugs were first launched in China, and they were all developed domestically. Most approvals received a priority review designation [129 (69.35%)], whereas conditional approval was much less granted [71 (38.17%)]. Over half of the approvals qualified for reimbursement through price negotiations [110 (59.14%)]. Many drugsattending the first four negotiations were approved by NMPA before 2015 and were therefore not included in the Listed Drug Database. As such, a significant portion of the reimbursed drugs in our sample [90 (81.82%)] obtained their reimbursement eligibility in the fifth and subsequent negotiations. Upon marketing, the reimbursed drugs took a median of 425 days (IQR, 272–614) to be listed on the formulary. The distributions of the reimbursement decision speed in terms of approval dates and negotiation rounds are depicted in Figures 1A, B, showing an accelerating trend of the time to reimbursement. It was noteworthy that the seventh round was originally scheduled to renew the formulary on 1 January 2023. Nonetheless, it had to be postponed for a period of 2 months due to the COVID-19 pandemic. The reimbursement decision speed associated with the seventh negotiation would be comparable to the previous round if the delay was excluded. Among all the approvals, the median time spent in the review process was 309 days (IQR, 258–394.5). As of 1 March 2023, the median follow-up was 475.5 days (IQR, 344–693).

**Table 1:** Descriptive characteristics of included approvals (*n* = 186).

**Characteristic**	***N* (%)**
**Registration class**	
NDA	102 (54.84)
BLA	84 (45.16)
**Approval class**	
Marketing authorization	93 (50.00)
New indication supplement	93 (50.00)
**Cancer site**	
Lung	44 (23.66)
Hematologic	39 (20.97)
Alimentary system	34 (18.28)
Breast	17 (9.14)
Genitourinary	12 (6.45)
Gynecological	8 (4.30)
Skin	8 (4.30)
Head and neck	7 (3.76)
Solid tumors	4 (2.15)
Thyroid	4 (2.15)
Other	9 (4.84)
**Approved year**	
< 2020	55 (29.57)
≥2020	131 (70.43)
**First approval region**	
China	74 (39.78)
The US	112 (60.22)
**Priority review**	
Yes	129 (69.35)
No	57 (30.65)
**Conditional approval**	
Yes	71 (38.17)
No	115 (61.83)
**Reimbursement**	
Yes	110 (59.14)
No	76 (40.86)
**Price negotiation winners (*****n*** = **110)**	
Round 2 (held in 2017)	1 (0.91)
Round 3 (held in 2018)	12 (10.91)
Round 4 (held in 2019)	7 (6.36)
Round 5 (held in 2020)	23 (20.91)
Round 6 (held in 2021)	35 (31.82)
Round 7 (held in 2023)	32 (29.09)
Reimbursement decision speed, median (IQR), days (*n* = 110)	425 (272–614)
Review times, median (IQR), days (*n* = 184)	309 (258–394.5)
Follow-up, median (IQR), days	475.5 (344–693)

BLA, biologics license application; NDA, new drug application; IQR, inter-quartile range.

### 3.2. Factors on the reimbursement decision speed

The results of the Cox regression analysis are summarized in Table 2, which included a sample size of 184. It was found that the drugs developed and first approved in China were associated with more chance of being covered by BMI (adjusted HR = 3.73, 95% CI 2.42 to 5.75; *P* < 0.001), implying a faster reimbursement decision speed for the domestic drugs. Moreover, NDA approvals were associated with a higher likelihood of receiving a positive reimbursement decision, in contrast to BLA approvals (adjusted HR = 2.14, 95% CI 1.35 to 3.39; *P* = 0.001). Different approval classes, the designations of priority review or conditional approval, the length of review duration, and the time trend were not directly correlated with the reimbursement decision speed of new drugs.

**Table 2:** Results of Cox regression on the reimbursement decision speed.

**Variable**	**Adjusted HR (95% CI)**	**Robust SE**	***P*-value**
**Approval class**			
Marketing authorization	1 (Reference)		
New indication supplement	1.01 (0.61–1.67)	0.26	0.972
**Registration class**			
BLA	1 (Reference)		
NDA	2.14 (1.35–3.39)	0.50	0.001
**Priority review**			
No	1 (Reference)		
Yes	1.38 (0.81–2.36)	0.38	0.232
**Conditional approval**			
No	1 (Reference)		
Yes	0.91 (0.59–1.42)	0.21	0.685
**First approval region**			
The US	1 (Reference)		
China	3.73 (2.42–5.75)	0.82	< 0.001
**Approved year**			
< 2020	1 (Reference)		
≥2020	1.22 (0.78–1.90)	0.28	0.386
Review times, days	1.00 (0.998–1.001)	< 0.01	0.945

HR, hazard ratio; CI, confidence interval; SE, standard error; BLA, biologics license application; NDA, new drug application.

It was interesting how the registration class would affect the qualification for reimbursement. Subsequently, we conducted the interaction analysis between the registration class and conditional approval pathway to investigate the effects of the conditional pathway among NDAs and BLAs (Table 3). It was observed that, in NDAs, regular approvals had a significantly larger probability of being adopted (adjusted HR = 2.15, 95% CI 1.13 to 4.08; *P* = 0.020) as compared to conditional ones, and in BLAs, conditional approvals were inversely related to the probability of being adopted than regular ones, despite the statistical insignificance (adjusted HR = 0.92, 95% CI 0.44 to 1.92; *P* = 0.816). The result of drugs first approved in China remained robust (adjusted HR = 3.73, 95% CI 2.41 to 5.76; *P* < 0.001). The interactions of the registration class with the priority review or approval class did not contribute more to explaining the variance of reimbursement decision speed, and further interaction analysis was hence not included.

**Table 3:** Interaction analysis for registration class and conditional approval.

**Variable**	Adjusted HR^*^(95% CI)	**Robust SE**	***P*-value**
**First approval region**			
The US	1 (Reference)		
China	3.73 (2.41–5.76)	0.83	< 0.001
**Interaction between registration class and conditional approval**			
BLA × regular approval	1 (Reference)		
BLA × conditional approval	0.92 (0.44–1.92)	0.35	0.816
NDA × regular approval	2.15 (1.13–4.08)	0.70	0.020
NDA × conditional approval	0.99(0.43–2.29)	0.42	0.989

^*^Adjusted for approval class, priority review, approved year, and review times. HR, hazard ratio; CI, confidence interval; SE, standard error; BLA, biologics license application; NDA, new drug application.

### 3.3. Sensitivity analysis

The results of the subgroup analysis for all drugs entering into the negotiations are presented in Supplementary Tables 2, 3. A total of 156 approvals were able to be in talks with the payer about reimbursement eligibility. The results demonstrated a consistent and strong positive correlation between drugs first approved in China and their time to reimbursement (adjusted HR = 3.16, 95% CI 2.03 to 4.93; *P* < 0.001). The interaction of registration class with conditional approval re-indicated the potential effect of early approval: NDA approvals that underwent the regular pathway were more likely to be considered reimbursable than ones through the conditional approval pathway (adjusted HR of regular NDA approval = 3.15, 95% CI 1.63 to 6.08; *P* = 0.001).

## 4. Discussion

This study provided the first investigation into the drug reimbursement decision speed in China, highlighting the increasingly improved access to novel oncology medications. Our findings demonstrated the efforts of the Chinese government to provide equitable healthcare of reasonable quality and to boost innovation in the domestic pharmaceutical industry. NHSA has specified the leading ideas of national price negotiations: prioritizing the major medical needs, filling the therapeutical area gaps, improving the drug formulary structure, and encouraging innovation (26). The increased likelihood for domestic new oncology drugs to access the national coverage plan was a result of the open-mindedness and strong support of the regulatory agency. With sufficient volume growth driven by reimbursement, a good sales increase can be achieved. For example, the domestic innovative antibody drug TYVYT^®^ (sintilimab injection) witnessed a 125.4% increase in sales in the first year of its access to BMI (27). The expanded revenue resulting from reimbursement is expected to incentivize domestic pharma companies to strive toward innovation, thereby enhancing their international competitiveness. However, the extent to which coverage for novel treatments by public plans contributes to innovation in the pharmaceutical industry awaits future studies.

Another important contributor to the faster reimbursement decision speed of domestic new drugs was the pricing advantage of Chinese pharma companies over global companies. In negotiations, overseas developers face a more intricate challenge of finding an appropriate price point: it should be capable of striking a balance between the anticipated rewards and the public interests; moreover, it is supposed to align with the company's global pricing strategy. However, the moderate ability of the Chinese population to pay can barely accomodate a relatively high price as in the developed world, while the low price set in China may undermine the price the global pharmaceutical industry can obtain in other regions. The concern about reference pricing troubles the domestic companies much less at the present stage. For many home-grown new drugs, there is no plan to expand their market beyond China in the near future, which, combined with the lower R&D costs in China, leads to a pricing advantage for domestic products. It should be noted that, nevertheless, the result does not indicate a reluctance on the part of the government to engage global drugmakers. Instead, China relies on them to provide access to ground-breaking medicines for patients in need; over 60% of the new oncology approvals that had been adopted via negotiations were developed by foreign companies. The price negotiation process is informed by health economic evaluations, whereby high prices that are commensurate with substantial clinical benefits may be deemed acceptable. Additionally, due to the different treatment landscapes between China and the developed countries, a novel drug may be valued higher in China when compared to the limited available therapies in this country. At present, “me-too” drugs still dominate in the domestic industry (28, 29), and in established drug classes, the low-price strategy can play some role in the negotiations, while first-in-class drugs can be immune to brand–brand competition absent followers. As the domestic industry grows its R&D capability and becomes more involved in the competition on international markets, the impact of different pricing strategies will be reduced. The favorable results observed for domestic oncology drugs need to be further confirmed, and the effects of the regulator's preference and the cost-effectiveness of products ought to be distinguished.

The reimbursement decision speed is relevant to an important tradeoff facing the payers but is little discussed in the scientific community. Reimbursing drugs with benefits in a timely fashion can facilitate patient access and sufficiently improve health outcomes, but paying for harmful or ineffective products will not only lead to a considerable waste of funds but also deteriorate patient health by preventing them from receiving adequate care. The new drugs with early market authorization, which are exemplified by conditional approvals, can risk more adverse events and a higher probability of being withdrawn, as many surrogate measures used as the basis for regulatory approval imperfectly correlate with the real benefits (30–32). These drugs with unproven benefits challenge payers with a tradeoff between accessibility and risk, of which the influence may be considerable. The accelerated approval program in the US—the analog to NMPA conditional approval—has cost Medicare more than $500 million for withdrawn indications lacking clinical benefits from 2017 to 2019 (33). Despite the fact that new payment methods have been devised to protect payers from the uncertainty of drug clinical benefits, their effects are found to be less desirable. The pay-for-performance strategy can only save <5% of the total drug cost due to the fact that continuous performance evaluations require costly infrastructure (34, 35). As such, the decision-making of payers would be constantly affected by the uncertainty around drug benefits.

Our study observed the effects of the expedited review pathway on reimbursement decisions, which yielded preliminary evidence on the tradeoff between accessibility and risk in the reimbursement process: regular approvals were more likely to be listed on the national formulary than conditional approvals. This tradeoff would exert a larger influence in budget-limited settings and discourage a prompt adoption decision for new technologies with conditional approvals. In addition to the risk of making the wrong reimbursement decision under uncertainty, the cost of reversal, e.g., the effort needed to change clinical practice after the reimbursement approval is withdrawn, may also be so sizable that the payer prefers to withhold purchases and wait for more information than immediately reimburse (36, 37). In addition to delayed adoption, reduced reimbursement or restricted coverage represent another more common manifestation of the tradeoff, which is likewise debated to be detrimental to patient access: insufficient reimbursement rates are unable to provide financial protection, while restricted coverage in the context of clinical studies tends to include fewer minorities and low-income individuals (38, 39). This tradeoff underscores the importance of appropriate risk management tools. It is critical that payers are tasked with managing risk by balancing accessibility against risk instead of minimizing risk. However, the risk management of payment for drugs with uncertain benefits remains quite inadequate in China. There is no payment policy related to conditionally approved new drugs, and this may be the important reason that the Chinese payer tends to postpone their reimbursements to mitigate the risks. A payment tool that is well designed for the Chinese context will play a key role in helping NHSA strike the delicate balance inherent in the reimbursement process.

The existing payment tools, mainly pay-for-performance and its refined variants (milestone payment, performance-based annuity, and outcome-based rebate) (40), have some key limitations precluding perfect risk management when used solely. As previously stated, they typically require quantifiable outcomes and costly continuous monitoring, which is not always feasible. Furthermore, as retrospective tools, performance-based installments or rebates are usually incapable of precisely quantifying each installment or rebate that is needed to fulfill risk management. The market authorization or reimbursement *per se* may damage the prospect of further evidence generation in post-approval periods as well (41). A prospective, quantifiable risk management tool in the reimbursement process is of much research value and will be a good supplement to refine access to medical innovations while also mitigating the loss of adopting inadequate products.

Whether a conditional approval pathway was designated or not in BLAs appeared to make little difference with regard to the reimbursement decision speed. This may be attributed, in part, to our rough dichotomization of the evidence uncertainty severity solely based on conditional approval designation. Among conditional approvals, the magnitude of uncertainty can vary. The points lie in the strength of surrogates, the trial design, and the way the conditional approval pathway is used (sometimes NMPA grants conditional approval to address ethnic sensitivity in the presence of efficacy evidence from foreign regions). Further in-depth study will help clarify the effects of evidence uncertainty.

This study has some limitations that should be stated. The first-round negotiation only contained three drugs that were all approved before 2015, and they were thus not included in our study. The findings did not present the situation for the first-round negotiation. The results did not reflect causal effects. The impact of the nationality of a drug developer and the expedited review pathways on the reimbursement decision making is worth of more research. Second, the database we utilize does not release review documents for all drugs, and there are hence some oncology approvals not included in the analysis. Third, beyond the study period, the reimbursement decision for some drugs may yet occur. Furthermore, as we have mentioned above, some features related to the drug's therapeutic value were not incorporated into the analysis. Apart from orphan designation, first-in-class is another valuable alternative; however, almost all domestic oncology drugs in our sample are me-too ones, due to which first-in-class is expected to have serious collinearity with the first approval region. The consequence of health technology assessment has been documented to impinge on reimbursement decisions (42–44), but the economic evaluations in price negotiations, including the cost-effectiveness analysis and budget impact analysis, are not available to the public.

## 5. Conclusion

The Chinese government has made headway in accelerating access to novel pharmaceuticals, and it is a positive signal for Chinese patients and drug developers across the globe. Domestic oncology new drugs seem to have faster reimbursement decision speed, which might be attributed to their advantages in drug pricing along with the regulatory support toward domestic industry growth and innovation. The higher reimbursement decision speed of regular approvals compared to conditional approvals suggests NHSA's concerns about the benefits of new drugs that lack robust evidence. Further research and regulatory efforts are required to help the payer better determine the balance between drug accessibility and risk.

## Data availability statement

The original contributions presented in the study are included in the article/Supplementary material, further inquiries can be directed to the corresponding author.

## Author contributions

XZ: conceptualization and design, acquisition, analysis, interpretation of data, statistical analysis, and drafting of the manuscript. YC: acquisition, statistical analysis, and review. All authors contributed to the article and approved the submitted version.
