# Supplementary material for: The reimbursement decision speed for oncology new drugs in China and its determinant factors
Source: Front Public Health. 2023 Oct 30;11:1207739. doi: 10.3389/fpubh.2023.1207739 (PMC10643204; doi:10.3389/fpubh.2023.1207739)
Supplement: Supplementary file 1 [file Table_1.docx]

Supplementary Material

The Reimbursement Speed for Oncology New Drugs in China and its Determinant Factors

**Xingyue ZHU^*^; Yang CHEN**

***Correspondence:** Xingyue ZHU: zhuxingyue@gmc.edu.cn.

# Supplementary Tables

Supplementary Table 1. Introductions of priority review and conditional approval in China.

| Feature | Priority review | Conditional approval |
| --- | --- | --- |
| Effective timing | Nov 2015 | Dec 2017 |
| Scope and requirements | Drugs with significant clinical benefits, AND:   1. Therapies in urgent needs, and innovative medications for major communicable diseases or rare conditions, OR 2. Pediatric new drug, new dosage and new strength, OR 3. Vaccines in urgent needs. | Innovative medications indicating for seriously debilitating or life-threatening conditions, AND:   1. Proves an effect on surrogate endpoints or intermediate endpoints that can reasonably predict clinical benefits, OR 2. Has encouraging early clinical data that can reasonably predict the superiority of clinical benefits over available therapies, OR 3. Orphan drugs that have been authorized outside of China. |
| Benefits | Abbreviate the review timeframe from 200 working days (for standard review) to 130 working days. | Early market entry before the clinical benefits materialize. |
| Commitment of sponsor | Not required | Post-marketing confirmatory trial to validate the clinical benefits anticipated based on the surrogate. |

Supplementary Table 2. The results of Cox regression on the reimbursement decision speed: the subgroup of drugs entering negotiations.

| Variable | Adjusted HR (95% CI) | Robust SE | P value |
| --- | --- | --- | --- |
| Approval class |  |  |  |
| Marketing authorization | 1 (Reference) |  |  |
| New indication supplement | 1.24 (0.78 to 1.99) | 0.30 | 0.358 |
| Registration class |  |  |  |
| BLA | 1 (Reference) |  |  |
| NDA | 2.76(1.71 to 4.45) | 0.67 | 0.000 |
| Priority review |  |  |  |
| No | 1 (Reference) |  |  |
| Yes | 1.10(0.65 to 1.84) | 0.29 | 0.728 |
| Conditional approval |  |  |  |
| No | 1 (Reference) |  |  |
| Yes | 0.91(0.58 to 1.43) | 0.21 | 0.693 |
| First approval region |  |  |  |
| The US | 1 (Reference) |  |  |
| China | 3.16(2.03 to 4.93) | 0.72 | <0.001 |
| Approved year |  |  |  |
| $<$2020 | 1 (Reference) |  |  |
| $\geq$2020 | 1.43(0.91 to 2.26) | 0.33 | 0.123 |
| Review times, days | 1.00(0.999 to 1.002) | <0.01 | 0.810 |

HR, hazard ratio; CI, confidence interval; SE, standard error; BLA, biologics license application; NDA, new drug application.

Supplementary Table 3. Interactions between registration class and conditional approval: the subgroup of drugs entering negotiations.

| Variable | Adjusted HR* (95% CI) | Robust SE | P value |
| --- | --- | --- | --- |
| BLA $\times$ regular approval | 1 (Reference) |  |  |
| BLA $\times$ conditional approval | 1.14 (0.53 to 2.44) | 0.44 | 0.733 |
| NDA $\times$ regular approval | 3.15 (1.63 to 6.08) | 1.06 | 0.001 |
| NDA $\times$ conditional approval | 0.72(0.31 to 1.70) | 0.31 | 0.455 |

*Adjusted for first approval region, approval class, priority review, approved year and review times.

HR, hazard ratio; CI, confidence interval; SE, standard error; BLA, biologics license application; NDA, new drug application.
